# Visible Light-Driven Micromotors in Fuel-Free Environment with Promoted Ion Tolerance

**DOI:** 10.3390/nano13121827

**Published:** 2023-06-08

**Authors:** Huaide Jiang, Xiaoli He, Ming Yang, Chengzhi Hu

**Affiliations:** 1 Shenzhen Key Laboratory of Biomimetic Robotics and Intelligent Systems, Department of Mechanical and Energy Engineering, Southern University of Science and Technology, Shenzhen 518055, China; 11930769@mail.sustech.edu.cn (H.J.); 11930743@mail.sustech.edu.cn (X.H.); 12149030@mail.sustech.edu.cn (M.Y.); 2 Guangdong Provincial Key Laboratory of Human-Augmentation and Rehabilitation Robotics in Universities, Southern University of Science and Technology, Shenzhen 518055, China

**Keywords:** micromotors, visible light, ion-tolerant, biocompatibility

## Abstract

Light-driven electrophoretic micromotors have gained significant attention recently for applications in drug delivery, targeted therapy, biosensing, and environmental remediation. Micromotors that possess good biocompatibility and the ability to adapt to complex external environments are particularly attractive. In this study, we have fabricated visible light-driven micromotors that could swim in an environment with relatively high salinity. To achieve this, we first tuned the energy bandgap of rutile TiO_2_ that was hydrothermally synthesized, enabling it to generate photogenerated electron-hole pairs under visible light rather than solely under UV. Next, platinum nanoparticles and polyaniline were decorated onto the surface of TiO_2_ microspheres to facilitate the micromotors swimming in ion-rich environments. Our micromotors exhibited electrophoretic swimming in NaCl solutions with concentrations as high as 0.1 M, achieving a velocity of 0.47 μm/s without the need for additional chemical fuels. The micromotors’ propulsion was generated solely by splitting water under visible light illumination, therefore offering several advantages over traditional micromotors, such as biocompatibility and the ability to operate in environments with high ionic strength. These results demonstrated high biocompatibility of photophoretic micromotors and high potential for practical applications in various fields.

## 1. Introduction

Micromotors capable of precise movement in small spaces to achieve specific tasks have garnered significant interest in recent years. With advancements in micro-nano processing technology, micromotors with increasingly complex designs have been developed and implemented in practical applications, including water remediation, drug delivery, biosensing, and targeted therapy [1,2,3,4,5,6]. These small devices can convert energy from different sources, such as chemical fuels, magnetic fields, or light fields, into mechanical motion, allowing them to navigate through intricate environments with high precision [7,8,9,10]. Light-driven electrophoretic micromotors (LEMs) are of particular interest due to their inexhaustible energy source, wireless control, fast response, and highly controlled motion trajectory [11,12,13]. Under light illumination, LEMs can interact with the surrounding environment by utilizing light energy to generate self-built electric fields or chemical gradient fields, which therefore provide driven force for micromotors [9]. The motion behavior of LEMs can be controlled by different directions, intensities, and wavebands of the incident light, allowing for complex light field control of LEMs. It has been demonstrated that light, as a tunable energy source, could power and control LEM motion precisely [14,15]. In addition, the moving velocity and the functionality of LEMs also largely depend on the properties of the semiconductor materials used for photo-electrochemical conversions, such as the energy bandgap, crystal phase, crystal plane orientation, and defect sites on the surface, etc. [16,17,18,19]. Zheng et al. modified TiO_2_/Si heterostructure-based nanotree micromotors with different photosensitizers. Due to the different light adsorption ranges of photosensitizers, the velocity of modified micromotors can be tuned by blue (475 nm) and red (660 nm) light illumination, which give micromotors excellent flexibility and high-level controllability [20]. Wolff et al. fabricated photocatalytic GaN/ZnO micromotors with an average velocity of 5.5 μm/s under UV light in an H_2_O_2_ solution. Notably, the fluorescence being generated by ZnO thin film makes micromotors promising for sensing applications [16]. By in situ growth of CdS quantum dots on C_60_ fullerene, Kochergin et al. demonstrated how the velocity of their fabricated CdS/C_60_ tubular micromotors could be tuned over the entire UV/Vis light spectra (320 to 670 nm). These micromotors hold considerable promise for designing smart micromachines that autonomously reconfigure their propulsion mode for on-demand operations, motion-based sensing, and enhanced cargo transportation [18].

Although LEMs offer distinct advantages over other types of micromotors, they still face a significant obstacle in practical applications, particularly in bio-related fields. The complex and dynamic external environment of LEMs, often characterized by various ions and biomolecules in solution, presents a challenge to their reliable and efficient operation. The high-concentration ions can quench the electrostatic driving force of most LEMs, making them unable to operate effectively [21]. To overcome this challenge, researchers have focused on developing ion-tolerant LEMs. Recently, Zhan et al. developed a polyaniline-coated micromotor that demonstrated enhanced ion tolerance under visible light illumination [22]. Sridhar et al. fabricated a poly (heptazine imide) (PHI) carbon nitride micromotor that could swim at high speeds in multicomponent ionic solutions with concentrations up to 5 M under 385 nm UV light illumination without the need for dedicated fuels [23]. While the addition of extra chemical fuels or the use of UV light can enhance the performance of LEMs, it may also have adverse effects, such as toxicity to living organisms and the photodegradation of materials used in micromotors. Therefore, the development of LEMs with excellent adaptability to complex environments while maintaining biocompatibility remains a significant challenge in this field.

In this study, we present a novel design of LEMs that can be propelled without the need for specific chemical fuels while exhibiting excellent phototaxis, even in ion-rich environments with NaCl concentrations as high as 0.1 M. To achieve this, we optimized the light absorption properties of rutile TiO_2_ by introducing oxygen vacancies and sulfur atoms, which extend its light absorption from UV to visible regions and reduce its energy bandgap to approximately 2.55 eV. We further increased the photocatalytic efficiency by decorating the TiO_2_ surface with platinum nanoparticles (Pt NPs) to prolong the lifetime of excited electron-hole pairs. To enhance ion tolerance, we coated the TiO_2_ surface with a layer of conductive polymer, polyaniline. Our newly developed micromotors exhibit obvious phototaxis under visible light (>400 nm) and can be propelled without the need for any additional chemical fuel. We believe that these micromotors represent a significant step forward in the development of LEMs, with promising practical applications.

## 2. Materials and Methods

### 2.1. Materials and Regents

Absolute ethanol (99.7 wt.%), ferrous sulfide (FeS) (Fe, 60–72 wt.%), sodium citrate dihydrate (99 wt.%), aniline (AR, ≥99.5 wt.%), ammonium persulfate (APS) (99.99 wt.%), and sodium borohydride (NaBH_4_) (98 wt.%) were purchased from Aladdin, Shanghai, China. Concentrated sulfuric acid (98 wt.%) and hydrochloric acid (40 wt.%) were purchased from Dongjiang Chemical Reagent Co., Ltd. (Dongguan, China). Tetrabutyl titanate (99 wt.%), oleic acid (85 wt.%), and chloroplatinic acid hexahydrate (H_2_PtCl_6_·6H_2_O) were obtained from Macklin, Shanghai, China. Deionized (DI) water with a resistivity of 18.2 MΩ/cm, produced in our laboratory, was used.

### 2.2. Instruments

The morphology of the micromotors was characterized using scanning electron microscopy (SEM, Zeiss Merlin (Oberkochen, Germany), operated at 5 kV and 100 pA). Electrochemical data were acquired using a Metrohm Autolab. Visible light illumination was provided by a Xenon lamp (CEL-HXUV300) equipped with a 400 nm cutoff filter, and a full-spectrum strong light power meter (CEL-NP2000) was used to measure the light intensity matched with the Xenon lamp (China Education Au-light Co., Ltd., Bejing, China). UV–visible DRS spectra were measured using a UV–vis spectrophotometer (Perkin Elmer LAMBDA 850, Sacramento, CA, USA). Transmission electron microscopy (TEM) images were taken by an FEI Talos. X-ray diffraction (XRD) spectra and electron paramagnetic resonance spectroscopy (EPR) were measured by Rigaku Smartlab and EMXPlus-10/12, respectively. An X-ray photoelectron spectrometer (XPS, PHI 5000 Versaprobe III) was used to investigate the binding energy of samples. The bath sonicator (360 W, 40 KHz, SB-5200DTD) was used for cleaning and dispersion of the samples. Motion videos were recorded using an optical microscope (Zeiss Axio Observer 5, Oberkochen, Germany) coupled with a 40× objective and a high-speed camera (Basler ace acA1920-150uc) using pylon view software. The camera had a resolution of 1544 × 1032 pixels, a frame rate of 30 fps, and a pixel size of 0.24 μm.

### 2.3. Procedures

#### 2.3.1. Doping the TiO_2_ Microspheres with Oxygen Vacancy and Sulfur

Firstly, rutile TiO_2_ microspheres featuring nanoneedle surface morphology were fabricated using hydrothermal methods [24]. Then, a mixture of 10 mg NaBH_4_ powder and 100 mg TiO_2_ was ground mechanically for half an hour, followed by heat treatment in a tube furnace at 350 °C for 6 h in an Ar gas atmosphere. Following naturally cooling to room temperature, the resultant powder was rinsed with 0.1 M HCl solution, ultrasonically cleaned multiple times, and dried in an oven. The reduced TiO_2_ (r-TiO_2_) powder was then placed into the bottom of a 2-necked flask, which was purged with Ar gas to eliminate air and maintained at 450 °C. Subsequently, H_2_S gas, generated by the reaction between FeS powder and 1 M HCl solution, was introduced into the flask for 1 h. After completion of the reaction, the flask was cooled to room temperature, and the powder was meticulously gathered, washed several times with DI water, and dried for later use.

#### 2.3.2. Pt Modification on r-S-TiO_2_

The method for Pt modification was adapted from existing literature with slight modifications [25]. Initially, 2 mL of H_2_PtCl_4_·6H_2_O (1 wt %) was dissolved in 50 mL of DI water followed by the addition of 100 mg of r-S-TiO_2_ powder. The mixture was then sonicated for 10 min to disperse the powder uniformly. Subsequently, the mixture was heated to 100 °C with continuous stirring at 200 rpm, and 3 mL of sodium citrate (1 wt %) was added. The solvent was then allowed to evaporate completely. Next, 50 mL of DI water was added, and the dispersion was purified through vacuum filtration at least 3 times to remove any unabsorbed Pt NPs. The r-S-TiO_2_@Pt powder was then separated from the solution using a centrifuge at 1500 rpm and calcined at 500 °C for 30 min in the air. The as-prepared sample was stored in an oven at 60 °C for further use.

#### 2.3.3. Preparation of r-S-TiO_2_@Pt/PANI Micromotors

Coating of PANI on micromotors was prepared by in situ chemical polymerization of aniline monomers onto r-S-TiO_2_ [26]. Initially, 20 mg of r-S-TiO_2_ and 0.1 mL aniline were dispersed in 20 mL of 1 M HCl solution and sonicated for 30 min. Subsequently, 2 mL of 1 M HCl solution containing 0.155 g APS was added to the above solution and sonicated for 4 h in an ice bath. Finally, the r-S-TiO_2_@Pt/PANI micromotors were collected and purified via vacuum filtration, followed by drying and storing in an oven for further use.

#### 2.3.4. Electrochemical Impedance Spectroscopy (EIS) Measurement

The procedure for preparing the working electrode for EIS measurement was as follows. First, 50 mg of pure TiO_2_, r-S-TiO_2_@Pt, or r-S-TiO_2_@Pt/PANI powders were compressed into tablets with a diameter of 4 mm and a thickness of approximately 0.5 mm using a tableting machine. Next, 1 side of the tablet was connected to a copper wire using silver paste. Both the wire and tablet were coated with epoxy resin, leaving only 1 side exposed. The working electrode, the Ag/AgCl electrode (−0.197 V vs. SHE), and Pt electrode were then immersed vertically into a 1 M H_2_SO_4_ solution. The DC potential was adjusted to the open circuit potential relative to the reference electrode (−0.197 V). The sinusoidal input signal was set to an amplitude of 10 mV, and the frequency varied from 1 × 10^6^ to 1 Hz.

#### 2.3.5. Motion Experiments

In a typical experiment, 10 μL of sample dispersions with a concentration of 0.1 mg/mL were dropped on a plasma pre-cleaned silicon wafer (10 × 10 mm). Visible light was illuminated with an incident angle of 30° to the dispersions. The dispersions were then subjected to visible light illumination with an incident angle of 30°. The tracking and velocity analysis of the micromotors were performed using Fiji software (version: 1.52n). Both auto-tracking and manual tracking techniques were employed to track the labeled micromotors and calibrate their velocities.

## 3. Results and Discussion

### 3.1. Fabrication Procedure and Work Mechanism of Micromotors

**Figure 1:**
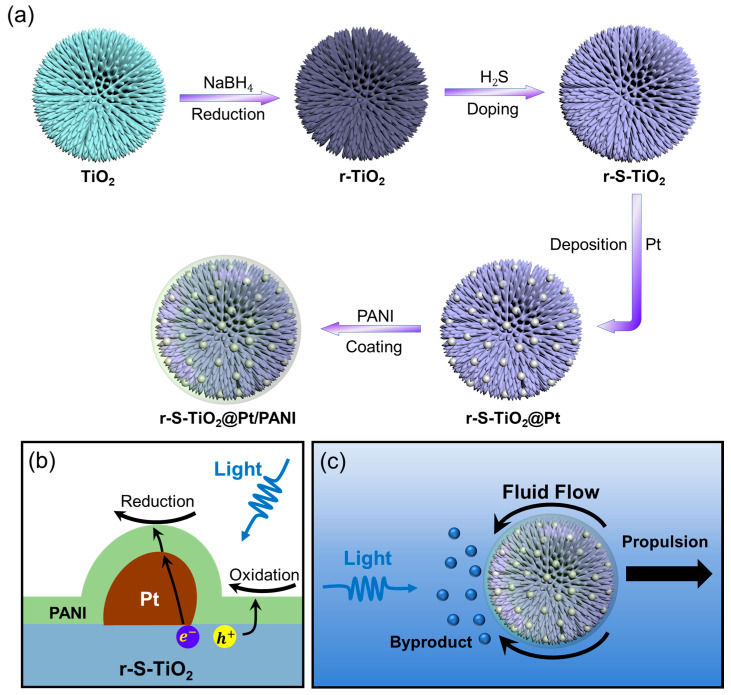
(**a**) Illustration of the fabrication process of TiO_2_@Pt/PANI micromotor; (**b**) diagram showcasing the photoelectrochemical reaction pathway that occurs on the surface of the micromotor; (**c**) propulsion mechanism of the micromotor under visible light illumination.

Figure 1a shows the schematic diagram of the doping process of TiO_2_ microspheres. Pure TiO_2_ was first reduced to r-TiO_2_ using NaBH_4_ as a reduction catalyst. Following that, sulfur (S) was doped into r-TiO_2_ through H_2_S gas flow at 500 °C to synthesize r-S-TiO_2_ microspheres. Direct S doping on rutile TiO_2_ is challenging, especially when trying to achieve an effective photocatalyst under visible light due to the relatively high energy barrier for S atoms to enter the TiO_2_ lattice. Some researchers have also processed S doping via the hydrothermal method, where both S and Ti precursors were put together and grown simultaneously to form S-doped TiO_2_ (S-TiO_2_) [27,28,29]. However, controlling the microstructure (shape, size, and surface morphology) of materials produced this way is difficult, making it unsuitable for batch fabrication. We first reduced TiO_2_ to r-TiO_2_ to enhance its visible light absorption capabilities and facilitate S doping due to the generation of oxygen vacancies [30]. S-doped TiO_2_ has been found to introduce a new energy band above the valence band, thereby reducing the energy band gap and making the material responsive to visible light [31]. 

Further improvement in the photoelectrochemical efficiency was accomplished by growing Pt nanoparticles on the surface of r-S-TiO_2_, which reduced the recombination efficiency of electron-hole pairs (Figure 1b). According to colloid and interface science theory, soft particles can maintain a certain ion tolerance in aqueous environments [32,33]. To enable the micromotors to operate in environments with relatively high ion concentrations, it was necessary to modify the surface of the micromotors with polymers that could endow them with ion tolerance as soft particles. In this study, the polymer coating on the surface not only acted as a barrier against ion penetration but also ensured the transport of photogenerated electron holes on the surface was not hindered. Consequently, conducting polyaniline (PANI) was selected as the coating polymer (Figure 1b). The working mechanism of r-S-TiO_2_@Pt/PANI micromotors is depicted in Figure 1c. Under visible light illumination, a redox reaction occurs on the illuminated side of the micromotors, leading to an asymmetric distribution of by-products on the micromotors, thereby driving the motion of micromotors.

### 3.2. Characterization of Surface Morphology and Composition of Micromotors

**Figure 2:**
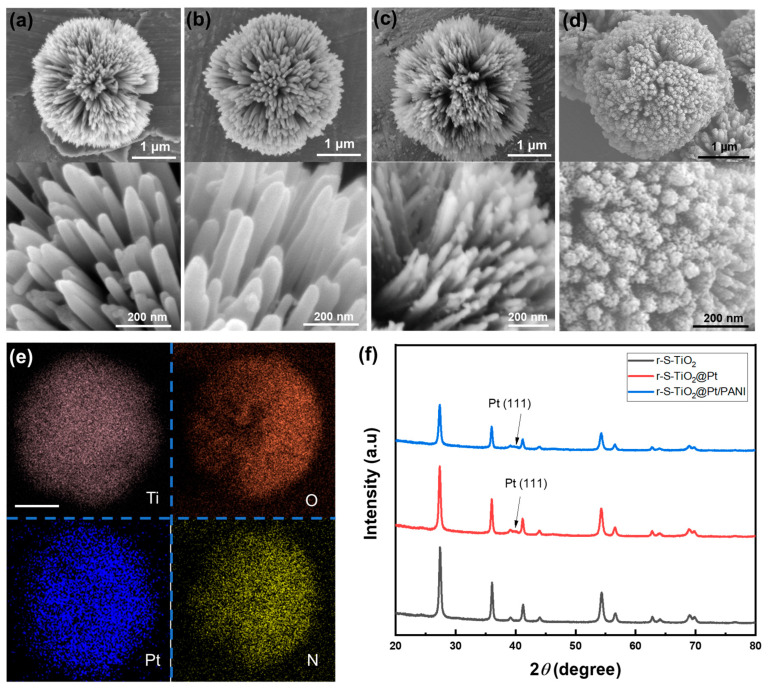
SEM images of (**a**) pure TiO_2_; (**b**) r-S-TiO_2_; (**c**) r-S-TiO_2_@Pt; and (**d**) r-S-TiO_2_@Pt/PANI micromotors; (**e**) EDX mapping of Ti, O, Pt, and N in r-S-TiO_2_@Pt/PANI micromotor. The scale bar is 1 μm; (**f**) XRD analysis of r-S-TiO_2_ (black), r-S-TiO_2_@Pt (red), and r-S-TiO_2_@Pt/PANI (blue).

Upon comparing pure TiO_2_ microspheres (Figure 2a) and r-S-TiO_2_ microspheres (Figure 2b), it was evident that the dual doping process did not lead to surface collapse, and the needle-like surface morphology remains intact. Both samples have similar diameters of approximately 3 μm, with a nanoneedle diameter of approximately 20 nm. The substantial specific surface area guarantees the availability of sample redox reaction sites, thereby enhancing the catalytic activity. Notably, the magnified SEM images at the bottom of Figure 2a,b reveal that the TiO_2_ needle arrays are free of impurities, which could adversely impact photocatalytic activity. Following the thermal decomposition of Pt on r-S-TiO_2_ (Figure 2c), small particles with diameters of approximately 10 nm are distinctly visible on the partially exposed surface of r-S-TiO_2_@Pt (bottom of Figure 2c). It is worth noting that excessive deposition of Pt may not only diminish the light absorption capacity of r-S-TiO_2_ but also create a recombination center for electron-hole pairs, resulting in a significant reduction in photocatalytic efficiency [34]. Consequently, maintaining an appropriate Pt to r-S-TiO_2_ ratio is crucial for the effective fabrication of the self-propelled micromotor. 

After coating PANI on r-S-TiO_2_@Pt, the surface of the micromotor is fully covered by PANI, resulting in a fluffy and porous surface (Figure 2d). To further confirm the Pt and PANI distribution on the micromotors, we conducted element mapping of Ti, O, Pt, and N of the micromotor by EDX, as shown in Figure 2e. The Pt and N elements align well with the shape and size of Ti and O, indicating the successful and uniform deposition of Pt and PANI on the micromotor surfaces. Figure 2f presents the XRD patterns. Pure TiO_2_ has previously been shown to exhibit a 100% rutile phase. After dual doping with oxygen vacancy and S, the phase of r-S-TiO_2_ was maintained as rutile (black curve), which was consistent with other reported findings. After the thermal deposition of Pt, the (111) plane of Pt was observed in both r-S-TiO_2_@Pt and r-S-TiO_2_@Pt/PANI samples (red and blue curves). However, the typical signal of PANI was not detected in the r-S-TiO_2_@Pt/PANI sample, possibly due to the amorphous structure of the PANI synthesized in this instance.

### 3.3. Characterization of Doping of TiO_2_

**Figure 3:**
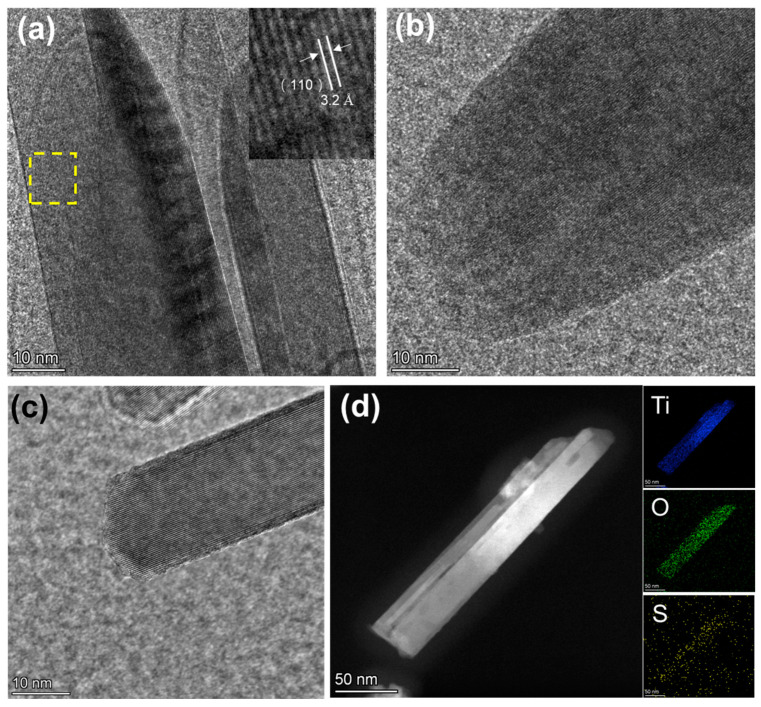
HRTEM images of (**a**) pure-TiO_2_. The insert image is an enlarged view of the yellow dashed area. (**b**) r-TiO_2_, and (**c**) r-S-TiO_2_; (**d**) EDS of r-S-TiO_2_ nanoneedle.

To demonstrate the successful doping of S into the TiO_2_ lattice, we first investigate the surfaces of pure TiO_2_, r-TiO_2_, and r-S-TiO_2_ using high-resolution transmission electron microscopy (HRTEM), as shown in Figure 3a–c. Prior to NaBH_4_ reduction, the surface of TiO_2_ appeared noticeably smooth, with the crystal faces of (110) exhibiting an interplanar spacing of 3.2 Å (Figure 3a). This observation further confirms the rutile phase of the as-fabricated TiO_2_. Following NaBH_4_ reduction, the surface of r-TiO_2_ became rough (Figure 3b). In Figure 3c, the presence of a core-shell structure is clearly evident after treating r-TiO_2_ with H_2_S gas. The core exhibits a crystal structure with a regular arrangement of atoms, while the shell displays a collapsed, disordered arrangement. This finding is consistent with the surface structure of other reported S-doped TiO_2_ materials [31]. Additionally, we captured a scanning transmission electron microscopy (STEM) image of a single r-S-TiO_2_ nanoneedle and conducted elemental mapping, as presented in Figure 3d. The distribution of titanium (blue) and oxygen (green) is in good agreement with the nanoneedle’s shape, while the presence of sulfur (yellow) is only faintly visible. We attribute this limited visibility to the low sulfur content, approximately 0.51 wt.%.

**Figure 4:**
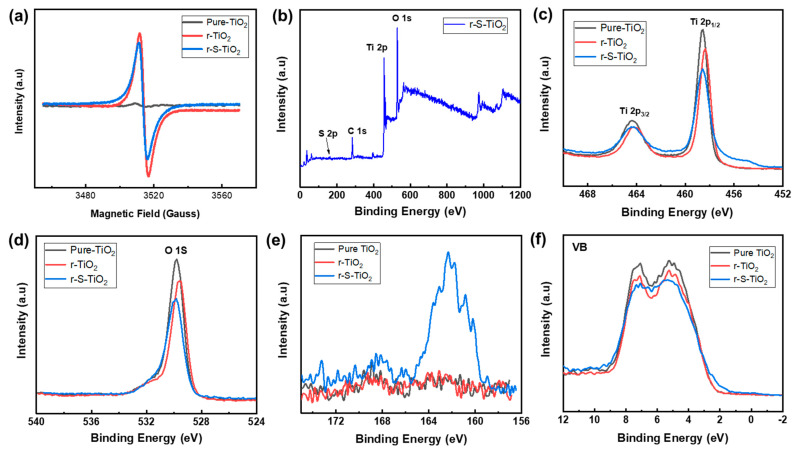
(**a**) Electron paramagnetic resonance of pure-TiO_2_ (black), r-TiO_2_ (red), and r-S-TiO_2_ (blue); (**b**) low-resolution XPS survey spectra of r-S-TiO_2_. High-resolution XPS spectra of (**c**) Ti 2p, (**d**) O 1s, (**e**) S 2p, (**f**) VB XPS of pure-TiO_2_ (black), r-TiO_2_ (red), and r-S-TiO_2_ (blue).

The reduction of NaBH_4_ can generate oxygen vacancies, potentially leading to the formation of Ti^3+^ ions on the sample surface. Consequently, O^2−^ ions can be adsorbed onto the surface of TiO_2_ [31]. Therefore, electron paramagnetic resonance (EPR) was conducted to further investigate the presence of Ti^3+^ states on the surface of r-S-TiO_2_. As depicted in Figure 4a, both r-TiO_2_ and r-S-TiO_2_ samples exhibit prominent signal peaks, while pure TiO_2_ shows negligible peaks. The peak intensity of r-TiO_2_ is higher than that of r-S-TiO_2_, primarily due to the incorporation of S into the TiO_2_ lattice, resulting in a reduced Ti^3+^ concentration. To further confirm the successful doping of S into r-TiO_2_, we performed an X-ray photoelectron spectroscopy (XPS) survey analysis of r-S-TiO_2_ (Figure 4b), revealing no obvious peaks other than those corresponding to O 1S, Ti 2p, and C 1s. This result confirms that no other impurity elements have been introduced. The peak intensity of Ti 2p1/2 (Figure 4c) and O 1s (Figure 4d) decreased as the reduction and S doping progressed, consistent with previous findings that suggest a decrease in peak intensity with a higher degree of reduction [35]. In Figure 4e, a broad S 2p signal ranging from 159.3 to 165.5 eV is observed in the r-S-TiO_2_ sample, with the peak located at approximately 162.3 eV. According to previous literature reports, S dopants are considered to substitute oxygen atoms in TiO_2_, indicating Ti-S bonding in the observed S 2p signal. In Figure 4f, the valence band XPS spectrum of r-S-TiO_2_ is compared with that of pure TiO_2_ and r-TiO_2_. While pure TiO_2_ and r-TiO_2_ exhibit a maximum energy edge of the valence band at approximately 2.07 eV, the valence band edge of r-S-TiO_2_ is shifted to approximately 1.5 eV, indicating that the valence band of r-S-TiO_2_ moves closer to the vacuum level at approximately 0.57 eV. 

### 3.4. Optical and Electronic Properties of Micromtors

**Figure 5:**
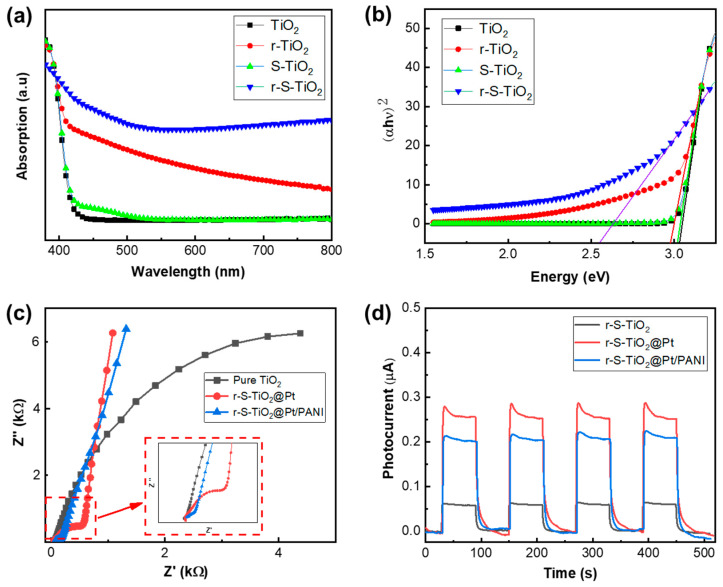
(**a**) UV–vis DRS spectroscopy of pure-TiO_2_ (black), r-TiO_2_ (red), and r-S-TiO_2_ (blue); (**b**) Tauc plot of the three samples derived from (**a**), the solid lines that intersect the horizontal axis are the tangents to the respective curves; (**c**) EIS spectroscopy of three samples: pure TiO_2_, r-S-TiO_2_@Pt, and r-S-TiO_2_@Pt/PANI; (**d**) photocurrent of r-S-TiO_2_, r-S-TiO_2_@Pt, and r-S-TiO_2_@Pt/PANI.

The optical properties of r-S-TiO_2_ were characterized utilizing UV–vis DRS spectroscopy, as shown in Figure 5a. It is observed that the sample of r-S-TiO_2_ significantly enhances the visible light absorption compared to both TiO_2_ and r-TiO_2_. S-TiO_2_ exhibits only a slight increase in the light range from 400 to 500 nm. As previously mentioned, without reducing TiO_2_, it is difficult to incorporate elemental S into the TiO_2_, resulting in negligible improvement in the efficiency of visible light. These observations were consistent with the absorption curves reported in other literature for S-doped TiO_2_ [36,37,38,39]. Based on the UV–vis DRS spectra, a Tauc plot was constructed, allowing the calculation of the energy bandgap for r-S-TiO_2_ to be approximately 2.55 eV. In contrast, the energy bandgaps for pure TiO_2_ and r-TiO_2_ were approximately 3.05 and 2.96 eV, respectively. The reduced energy bandgap of r-S-TiO_2_ signifies its capacity to harness visible light, generate electron-hole pairs, and participate in redox reactions, thereby driving photoelectrochemical reactions.

After the application of PANI onto r-S-TiO_2_@Pt, the charge transfer resistance (*R_ct_*) of r-S-TiO_2_/Pt@PANI was measured using electrochemical impedance spectroscopy (EIS) and compared with those of r-S-TiO_2_ and r-S-TiO_2_@Pt, as shown in Figure 5c. The presence of Pt in r-S-TiO_2_@Pt and r-S-TiO_2_@Pt/PANI samples significantly reduced *R_ct_* in comparison to pure TiO_2_. Furthermore, the continuous deposition of PANI resulted in a further decrease in *R_ct_* of r-S-TiO_2_@Pt/PANI (approximately 407 Ω), which was 22.5% of that of r-S-TiO_2_@Pt. Notably, the slope of the curves for r-S-TiO_2_@Pt in the low-frequency region was slightly higher than that of r-S-TiO_2_@Pt/PANI. This effect was attributed to the hindering effect of the polymer on the diffusion of particles. To investigate the photocatalytic activity of micromotors under visible light illumination, we measured the photocurrent of r-S-TiO_2_, r-S-TiO_2_@Pt, and r-S-TiO_2_@Pt/PANI samples by using an electrochemical workstation at a visible light intensity of 1.6 W/cm^2^ (Figure 5d). After Pt deposition on micromotors, the photocurrent intensity increased by approximately 5.3 times compared to r-S-TiO_2_. However, the r-S-TiO_2_@Pt/PANI sample showed a decrease of almost 75.5 % relative to r-S-TiO_2_@Pt. We speculated that this was mainly due to the partial absorption of light by PANI, which weakened the absorption of light by TiO_2_.

### 3.5. Motion Behavior of Micromotors in Pure Water 

**Figure 6:**
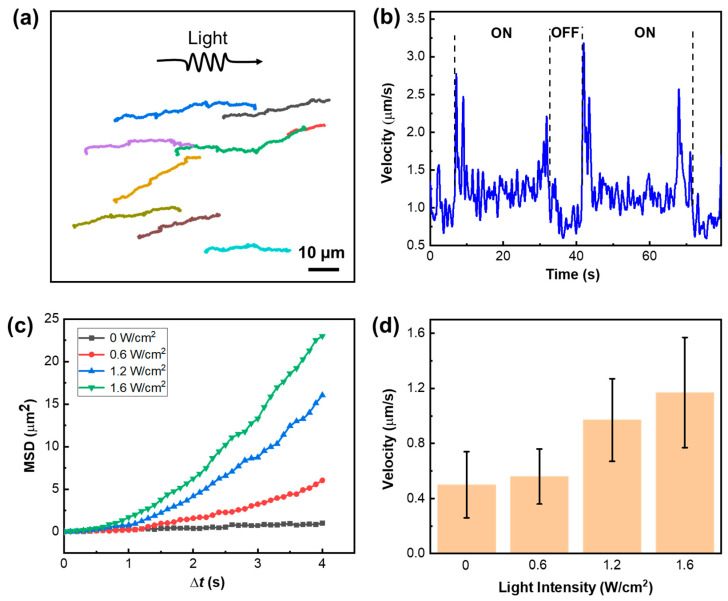
(**a**) Motion trajectory of r-S-TiO_2_@Pt/PANI micromotors under 1.6 W/cm^2^ visible light (>400 nm) illumination, note that lines of different colors represent the trajectory of different micromotors. (**b**) velocities of r-S-TiO_2_ micromotors in ON/OFF states of visible light; (**c**) the MSD curves of micromotors under different light intensities; (**d**) histogram of micromotor velocities under different light intensities.

We first characterized the motion behavior of r-S-TiO_2_@Pt/PANI micromotors in a pure water environment under visible light illumination (Video S1). The motion trajectories of the micromotors are captured and presented in Figure 6a during approximately 60 s of 1.6 W/cm^2^ light illumination. The negative phototactic behavior of the micromotors is demonstrated by their movement away from the light source, and their average velocity is calculated to be approximately 1.65 μm/s. It is widely recognized that UV photons possess higher energy compared to visible photons, thereby leading to a more efficient excitation of electron-hole pairs during photoelectrochemical reactions. In addition, although doped rutile has visible light catalytic activity, its catalytic activity under visible light is lower than that under UV. As a result, the velocities of r-S-TiO_2_@Pt/PANI micromotors under visible light were lower than those in our previous work, which were driven under UV light illumination. In Figure 6b, the obvious step change in velocity under the on/off states of light was plotted, which indicates that the directional motion of the micromotor was influenced by the photoelectrochemical reaction. To gain further insight into the working mechanism of the micromotors, we calculated the mean squared displacement (MSD) under various intensities of visible light (Figure 6c). The quadratic curves of MSD suggested the electrophoretic/electroosmotic motion of the micromotors. The present micromotor has an isotropic structure; thus, when unidirectional light is irradiated on its surface, only the illuminated side produces photoelectrochemical reactions and generates by-products, which leads to an asymmetric distribution of by-products between the light-facing and the back-light side. Therefore, the by-product provides propulsion force for micromotors’ directional movement mainly through electrophoretic/electroosmotic effects [24,40]. The velocity of micromotors vs. light intensity was also plotted in Figure 6d. As the incident light intensity increased, the velocities were noticeably enhanced, indicating the direct influence of light intensity on the micromotors’ velocity. Note that the velocity of micromotors is not obvious to see when lower visible light intensity is applied. To obtain effective motion behavior, we chose the value of light intensity to be 1.6 W/cm^2^, which is comparable to those micromotors with visible light photocatalytic activity [40,41].

### 3.6. Motion of Micromotors in Different Concentrations of NaCl Solutions

**Figure 7:**
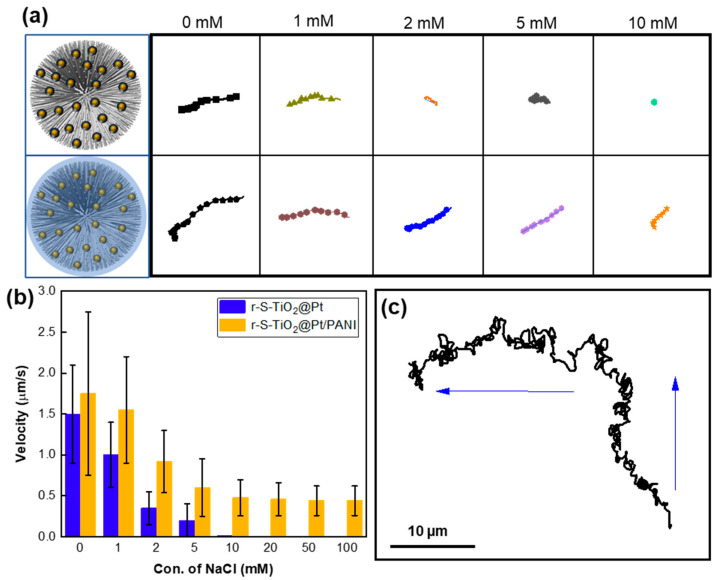
(**a**) Motion trajectory of r-S-TiO_2_ micromotors at different NaCl concentrations relative to r-S-TiO_2_; (**b**) velocities of micromotors vs. different concentrations of NaCl; (**c**) trajectory of micromotor under different light illumination direction (blue arrow).

The motion of light-driven micromotors is significantly influenced by the salinity in the solution. As the concentration increases, the speed of the micromotors tends to decrease, while their trajectory becomes more erratic [22,42]. When polyelectrolytes with ion-permeable surface layers are assembled on a colloidal particle, the electrical diffuse double layer formed between the particle and electrolyte solution exists not only at their interface but also within the surface charge layer of polyelectrolytes. This plays a vital role in maintaining the non-zero electrophoretic mobility of micromotors at higher salinity levels [42]. After coating PANI on r-S-TiO_2_@Pt, the resulting micromotors demonstrated favorable ion-tolerant characteristics. In Figure 7a, the moving trajectory of micromotors between r-S-TiO_2_@Pt and r-S-TiO_2_@Pt/PANI was compared in NaCl solutions of different concentrations, ranging from 0 to 10 mM. As the ion concentration increased, the motion displacement of the r-S-TiO_2_@Pt micromotor decreased significantly and eventually reached zero at an ion concentration of 10 mM due to the quenching of the electric double layer by ions. However, the r-S-TiO_2_@Pt/PANI micromotor still retained 29.7% of its displacement in 10 mM NaCl solution compared to that in 0 mM NaCl solution.

We also calculated the velocities at increasing ion concentrations (Figure 7b), where the velocity of the r-S-TiO_2_@Pt micromotor quickly decreased from 1.5 μm/s to 0 when the ion concentration reached 10 mM. In contrast, the velocity of the r-S-TiO_2_@Pt/PANI micromotor remained at 0.47 μm/s even in 0.1 M NaCl solution. Thus, the as-prepared micromotors exhibited a significant improvement in ion tolerance in NaCl solutions as high as 0.1 M. It is worth noting that r-S-TiO_2_@Pt micromotors exhibited positive phototaxis in motion while r-S-TiO_2_@Pt/PANI micromotors exhibited negative phototaxis behavior. The coating of PANI, which contains abundant amino groups, changes the zeta potential of the micromotor, which is a core factor influencing the motion behaviors of LEMs as our previous work has demonstrated [24]. Furthermore, we investigated the controllability of micromotors, as shown in Figure 7c. The trajectory of the micromotors was recorded as the light direction changed within two minutes of illumination. The micromotor maintained negative phototaxis motion behavior even at high ion concentrations, proving the effective control of micromotors by illumination direction.

## 4. Summary

In this work, we successfully tuned the bandgap of rutile TiO_2_ from 3.0 to 2.55 eV through oxygen vacancy and sulfur doping, making it responsive to visible light. After modifying its surface with Pt and PANI, the as-prepared r-S-TiO_2_@Pt/PANI micromotors displayed distinct negative phototactic motion behavior with a velocity of 1.65 μm/s under 1.6 W/cm^2^ visible light illumination in a pure water environment. More importantly, the polyelectrolyte PANI on the surface significantly enhanced its ion tolerance, enabling it to swim in a 0.1 M NaCl solution. Designing LEMs to adapt to high-salinity environments is challenging. The velocity of our TiO_2_-based micromotors can still be improved via many approaches. Introducing metal (e.g., Fe, Co, Ni, Ag) or non-metal (e.g., N, C, F) dopants into the TiO_2_ lattice can modify its electronic structure, reduce the bandgap, and enhance visible light absorption. Creating heterojunctions between TiO_2_ and other semiconductors (e.g., ZnO, CdS, WO_3_, BiVO_4_) can facilitate charge separation and reduce the recombination rate of photogenerated electron-hole pairs, thereby improving photocatalytic performance. Surface modification with noble metals (e.g., Au, Pt, Pd) or co-catalysts (e.g., graphene, carbon nanotubes) onto the surface of TiO_2_ can serve as electron sinks or provide additional active sites, promoting charge separation. Tailoring the morphology (e.g., nanoparticles, nanorods, nanosheets, nanofibers) and crystal structure (e.g., anatase, rutile, brookite) of TiO_2_ can influence its surface area, crystallinity, and light absorption properties. Recently, it has been found that incorporating plasmonic nanostructures (e.g., Au, Ag nanoparticles) into TiO_2_ can enhance its light absorption through the generation of localized surface plasmon resonance and improve photocatalytic activity under visible light. By exploring these strategies, the photocatalytic activity of electrophoretic TiO_2_-based micromotors demonstrates significant potential for a variety of applications, including water and air purification, energy conversion, and environmental remediation.

## Data Availability

Data sharing is not applicable to this article.

## Supplementary Materials

The following supporting information can be downloaded at: https://www.mdpi.com/article/10.3390/nano13121827/s1; Video S1: r-S-TiO_2_@Pt/PANI micromotors swimming in fuel-free environment.
